# Voluntary exercise preserves visual function and reduces inflammatory response in an adult mouse model of autosomal dominant retinitis pigmentosa

**DOI:** 10.1038/s41598-024-57027-9

**Published:** 2024-03-23

**Authors:** Katie L. Bales, Austin M. Karesh, Kelleigh Hogan, Alicia S. Chacko, GianMarco L. Douglas, Andrew J. Feola, John M. Nickerson, Alyssa Pybus, Levi Wood, Jeffrey H. Boatright, Machelle T. Pardue

**Affiliations:** 1 Atlanta VA Medical Center for Visual and Neurocognitive Rehabilitation, Decatur, GA USA; 2 Wallace H. Coulter Department of Biomedical Engineering, Georgia Institute of Technology, Atlanta, GA USA; 3 Department of Ophthalmology, Emory University, Atlanta, GA USA; 4 George W. Woodruff School of Mechanical Engineering and Parker H. Petit Institute for Bioengineering and Bioscience, Georgia Institute of Technology, Atlanta, GA USA; 5 Department of Ophthalmology, Emory University, 1365B Clifton Road NE, Rm. 2600, Atlanta, GA 30332 USA

**Keywords:** Retina, Mechanisms of disease

## Abstract

Whole-body physical exercise has been shown to promote retinal structure and function preservation in animal models of retinal degeneration. It is currently unknown how exercise modulates retinal inflammatory responses. In this study, we investigated cytokine alterations associated with retinal neuroprotection induced by voluntary running wheel exercise in a retinal degeneration mouse model of class B1 autosomal dominant retinitis pigmentosa, I307N *Rho*. I307N *Rho* mice undergo rod photoreceptor degeneration when exposed to bright light (induced). Our data show, active induced mice exhibited significant preservation of retinal and visual function compared to inactive induced mice after 4 weeks of exercise. Retinal cytokine expression revealed significant reductions of proinflammatory chemokines, keratinocyte-derived chemokine (KC) and interferon gamma inducible protein-10 (IP-10) expression in active groups compared to inactive groups. Through immunofluorescence, we found KC and IP-10 labeling localized to retinal vasculature marker, collagen IV. These data show that whole-body exercise lowers specific retinal cytokine expression associated with retinal vasculature. Future studies should determine whether suppression of inflammatory responses is requisite for exercise-induced retinal protection.

## Introduction

Mutations in genes encoding retinal trafficking proteins often manifest as blinding diseases, such as retinitis pigmentosa (RP). RP is a heterogeneous group of hereditary disorders that lead to the progressive loss of retinal function. RP is one of the most inherited blinding diseases, affecting about 1.5 million people globally, with roughly 25% of these cases being autosomal dominant retinitis pigmentosa (adRP)^1–4^. To date, 24 genes have been associated with adRP, (RetNet; final version, dated October 7, 2022) and over 1000 mutations have been reported in these genes^5^. Clinical features include a reduction in the peripheral visual field, leading to tunnel vision and eventual progression to total blindness. Disease onset typically begins in the early teenage years, and severe visual impairment occurs between the ages of 45 and 60^6^. Although therapeutic strategies targeting specific mutations are being developed^2,7,8^, clinical trials and retrospective studies suggest that exercise may have preventative and rehabilitative effects in patients with retinal degenerative diseases, including inherited retinal degenerations^9–12^.

Recent studies have shown physical exercise to be an effective, non-invasive strategy to prevent and halt neurodegenerative disease progression^11,13–15^. For the past decade, our group has investigated the neuroprotective effects of exercise in several animal models of retinal disease. We have found exercise to be associated with reduced photoreceptor cell death, reduced retinal pigment epithelium (RPE) stress, conserved retinal structure, preserved visual function, and increased neuroplasticity in retinal astrocytes^16–22^. Recently, we determined voluntary running wheel exercise preserved retinal function, morphology and retinal pigmented epithelial (RPE) integrity in aged (10–20-month-old) I307N *Rho* mice^18^. This model has high translational relevance, because it specifically mimics several phenotypes associated with class B1 adRP mutations, such as slower disease progression following induction, focal rod degeneration, and the initiation of innate and acquired retinal immune responses^23,24^. Immune dysregulation is one of the key mechanisms underlying retinal degenerative disease pathogenesis^25^. Using cytokine knockout animal models, studies have shown that chronic stress in a deficient innate immune system may tip the balance of the cytokine network towards an exacerbated immune response, resulting in tissue and cellular damage^26–28^. It is currently unknown how whole-body exercise affects retinal immune responses and cytokine signaling networks, specifically in adRP. Our study provides insight for future methods and treatments to halt the progression of inherited retinal degenerations. In this study, we sought to investigate the effects of voluntary exercise on retinal structure, function, and retinal cytokine response in younger (3–4 month-old) I307N *Rho* mice.

## Methods

### Animals

**Figure 1:**
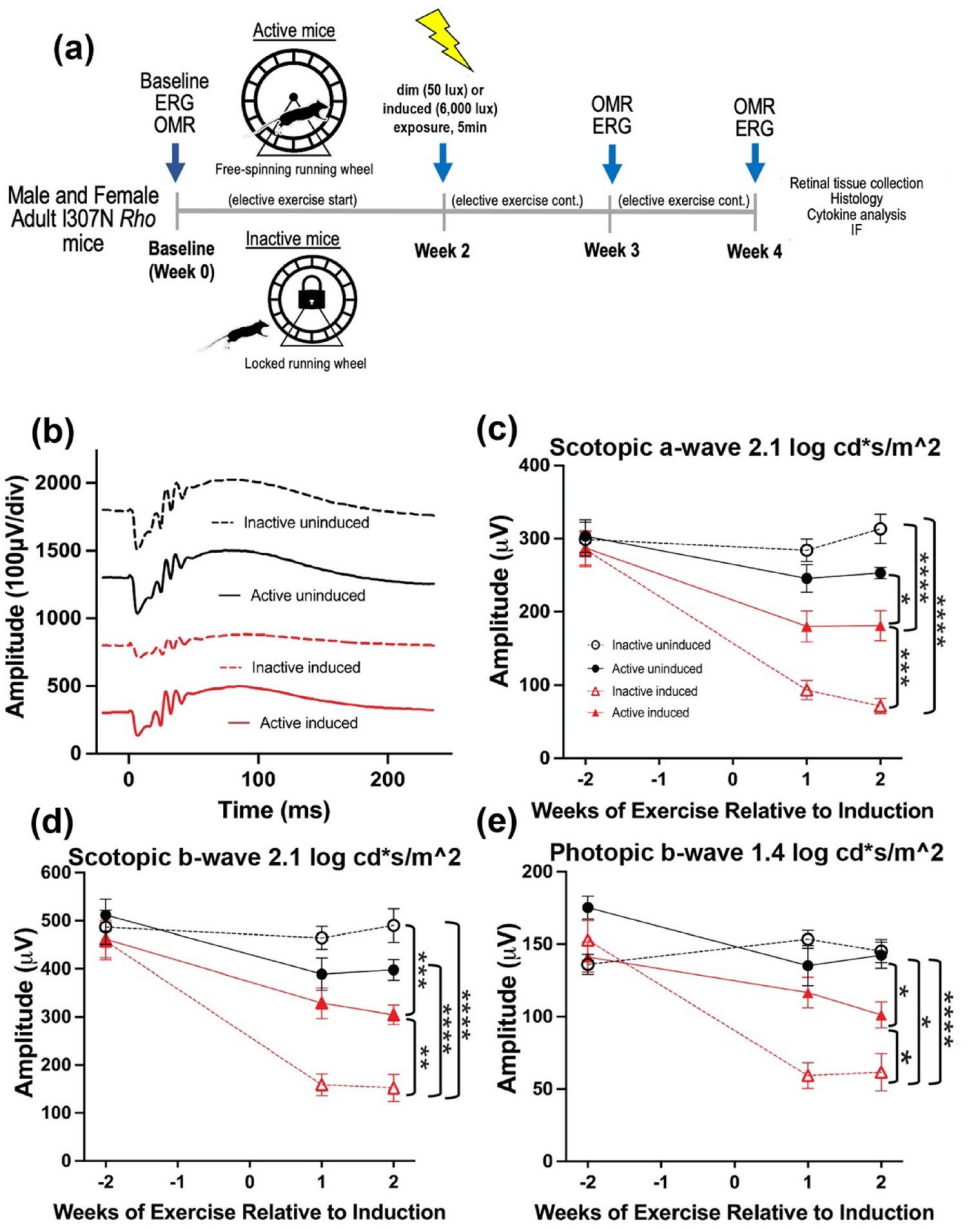
Voluntary running wheel exercise preserved retinal function in induced I307N *Rho* mice. To test the effects of voluntary exercise on retinal and visual function, we performed electroretinography (ERGs) and optomotor response (OMRs) measurements at baseline, 2- and 4-weeks post light induction (**a**). To ensure exercise-induced retinal neuroprotection was achieved as demonstrated in our labs previously, electroretinography (ERG) recordings were performed to non-invasively measure retinal function. Representative ERG waveforms are shown from dark-adapted stimuli (2.1 log cd s/m^2^; **b**). Active induced mice had significant preservation of a-wave (**c**), b-wave (**d**) and photopic b-wave amplitudes (**e**) compared to inactive induced mice. a- and b-waves show rod photoreceptor and inner retinal function, respectively at 2-weeks post light induction. The photopic ERG measures cone photoreceptor function. Two-way ANOVA with Tukey’s multiple comparison analysis was performed. N = 11–13 per group, *p < 0.05, **p < 0.01, ***p < 0.001, ****p < 0.0001. Values are mean ± SEM. *IF* immunofluorescence.

All animal procedures were approved by the Atlanta VA Institutional Animal Care and Use Committee, conform to the Association for Research in Vision and Ophthalmology (ARVO) Statement for the Use of Animals in Ophthalmic and Vision Research, and all methods are reported in accordance with ARRIVE guidelines. A colony of I307N *Rho* mice was established and maintained at Emory University by breeding homozygous I307N *Rho* mice (gift of Dr. Patsy Nishina of the Jackson Laboratory, Bar Harbor, ME; RRID: IMSR_JAX:030638; MGI: J:159523) to produce heterozygous progeny for use in experiments^29,30^. (*N.B*.: The mice from Dr. Nishina originated from a two-generation backcross mating scheme using chemically mutated F1 hybrid 129/SvJae × C57BL/6J embryonic stem cells, followed by a minimum of five backcrosses onto the C57BL/6J background)^29^. Adult male and female (3–4 months-old, n = 11–16 per group) heterozygous I307N *Rho* mice were then transferred to the Atlanta VA and housed under a 12-h light/dark cycle (06:00–18:00 h). During the light cycle, light levels measured at the bottom of the mouse cages approximately 50 lx. Mice had access to standard mouse chow (Teklad Global 18% Protein Rodent Diet 2918, Irradiated, Rockville, MD) and water ad libitum. Mice were placed in single housing cages with low-profile running wheels (Med-Associates, Inc.; St. Albans, VT) that were either functional (active) or locked (inactive; see schematic of experimental timeline in Fig. 1a). Mice had continuous access to wheels for the entirety of the experiment, with the exception of when retinal and visual functional testing was performed and during light induction (detailed below). Through use of voluntary running wheels, we are able to model running patterns similar to natural running behavior in mice, providing a non-stress environment comparatively to forced exercise such as treadmill running^31^. When given access to running wheels, mice have been reported to run a range between 4 and 20 km per day with a total activity time of roughly 3 to 7 h a day^31^. Following testing at baseline, light exposure, and testing at 1- and 2-weeks post-light induction, mice were then transferred back to maintenance housing with active or inactive running wheels. At the end of 4-weeks, mice were euthanized via cervical dislocation, eyes were enucleated for histology and immunohistochemistry, and retinal extract was collected for a multiplexed cytokine immunoassay.

### Induction of the I307N* Rho* degeneration

Animals were dark adapted overnight and atropine eye drops (0.2% diluted from 1% atropine ophthalmic solution, Akorn Inc., Lake Forest, IL; diluted with Refresh Tears (Allergan, Irvine, TX)) were administered twice, with the last eye drops applied 30 min before toxic light exposure. To induce I307N *Rho* degeneration, animals were individually housed in shoebox containers with a white light emitting diode (LED) light panel (LED500A; Fancierstudio, Hayward, CA) fitted to a standard mouse cage as previously described^19^. Mice were exposed to either bright light (6000 lx, induced) to induce retinal degeneration or dim light (50 lx, uninduced) for 5 min. Room and light box temperatures were closely monitored to ensure animal welfare. Light induction occurred between 10 and 11 AM. Following light induction, mice were returned to their home cages under normal lighting conditions for the remainder of the experiment.

### Electroretinography (ERG)

Retinal function (n = 11–13 per group) was measured with a commercial electroretinography (ERG) system (Bigshot; LKC Technologies, Gaithersburg, MD) as previously described by our group^16,19^. After overnight dark adaptation, mice were anesthetized (ketamine [80 mg/kg]/xylazine [16 mg/kg]). All procedures were performed under dim red light. The pupils were dilated (1% tropicamide; Alcon Laboratories, Ft. Worth, TX) and corneas were anesthetized (1% tetracaine; Alcon Laboratories, Ft. Worth, TX). Body temperature was maintained with a heating pad (ATC 1000; World Precision Instruments, Sarasota, FL) for the duration of the session. The ERG protocol consisted of a ten-step series of full-field flash stimuli produced by a Ganzfeld dome under both dark-adapted scotopic conditions (− 3.0 to 2.1 log cd s/m^2^) to test rod and rod/cone pathways and light-adapted photopic conditions to test cone pathway function. (2.0 log cd s/m^2^ presented as 6.1 Hz flicker with a 30 cd/m^2^ background light). Custom gold-loop wire electrodes were placed on the center of each eye through a layer of 1% methylcellulose to measure the electrical response of the eye to each flash. Both reference and ground platinum needle reference electrodes (1 cm; Natus Medical Incorporated, Pleasanton, CA) were inserted subcutaneously in each cheek and the tail, respectively. Once ERG was completed, mice were given an IP injection of atipamezole (1 mg/kg; Antisedan, Zoetis, Parsippany, NJ) to counteract the effects of xylazine^32^, administered saline eye drops and allowed to recover on a heating pad (37 °C) before being returned to housing. ERG responses from both eyes were averaged together.

### Optomotor response (OMR)

A virtual OMR tracking system was used to measure visual function in all experimental groups (n = 11–16 per group; OMR; OptoMotry®; Cerebral Mechanics Inc., Lethbridge, AB, Canada) as previously described by our group and others^33–36^. Mice were placed on a circular platform in the center of a virtual reality chamber composed of four computer monitors, which present vertical sine wave gratings revolving at a speed of 12 deg/s. A video camera monitored the animals’ behavior in real time throughout the experiment. The presence or absence of reflexive movements by the mouse’s head were noted as it tracked the rotating gratings moving either in a clockwise or counter-clockwise direction. When mice became distracted, their attention was regained by gently tapping the instrument. OptoMotry® uses a staircase paradigm to automatically calculate spatial frequency and contrast sensitivity thresholds. To evaluate spatial frequency, contrast was held constant at 100% while spatial frequency gratings started with a frequency of 0.042 cyc/deg and adjusted over time with the presence or absence of head movements. To assess contrast sensitivity, spatial frequency was held constant at the peak of the contrast sensitivity curve at 0.064 cycles per deg (c/d), while contrast began at 100% and adjusted over time. The contrast sensitivity reported here was calculated as a reciprocal of the Michelson contrast from the screen’s luminance (max + min/max − min), as previously described^37^. The OMR was assessed by a masked observer. The spatial frequency and contrast sensitivity thresholds were determined after three positive responses and calculated as the average of the reported values for both eyes and were compared across time points.

### Histology and morphometrics of ocular sections

For histological analyses, eyes were enucleated and then immersion-fixed in 4% paraformaldehyde for 2 h at room temperature and rinsed with 0.1 M phosphate buffer. Posterior eyecups were processed through a graded alcohol series and embedded in plastic resin (Embed 812/DER 736, Electron Microscopy Science). Using an ultramicrotome (Reichert Ultracut, Leica, Inc., Buffalo Grove, IL) with a histo-diamond knife, sections (0.5 μm) were cut through the superior to inferior retina bisecting the optic nerve. Plastic embedded retinal sections were stained with 1% aqueous toluidine blue (Millipore Sigma, Burlington, MA) and imaged using a Leica DMLB microscope with a 40 × N Plan brightfield objective (Leica, Inc., Buffalo Grove, IL). Retinal spidergrams were constructed by plotting the number of photoreceptor nuclei present in the outer nuclear layer (ONL) and cone photoreceptor quantifications as a function of position in the retina relative to the optic nerve. ONL and cone nuclei were manually counted using Adobe Photoshop (24.0.0 release) within 100-μm-wide segments spaced at 250, 500, 750, 1000 and 1250 μm from the optic nerve head in the superior and inferior directions within whole retinal sections (n = 8 per group, 3 retinal sections quantified per animal).

### Immunofluorescence

Eyes were fixed in methanol acetic acid, dehydrated, embedded in paraffin, and sectioned through the sagittal plane on a microtome (5 μm). Sections containing the optic nerve were selected for staining to ensure that consistent regions were examined between animals. The slides were deparaffinized across five Coplin jars with 100 mL of xylene for 2 min each, consecutively. Then the slides were rehydrated in a series of 100 mL ethanol solutions for 2 min each: 100%, 90%, 80%, 70%, 60%, and 50%. Following rehydration, retinal sections were blocked for 30 min at room temperature in 5% normal donkey serum in PBS with 0.01% sodium azide and 0.3% Triton X-100. Primary antibodies were diluted in 5% normal donkey serum in PBS with 0.01% sodium azide and incubations were performed overnight at 4 °C using KC monoclonal antibody (48415, Invitrogen, Waltham, MA, 1:100 dilution), IP-10 monoclonal antibody (sc-374092, Santa Cruz Biotechnology, Dallas, TX, 1:100 dilution), Gt X Collagen Type IV antibody (AB769, Millipore Sigma, Burlington, MA, 1:100 dilution) and cone arrestin (15282, Millipore Sigma, Burlington, MA, 1:100 dilution). Secondary antibodies were diluted in PBS and incubations were performed for 1 h at room temperature in the dark (Alexa Fluor® 647 donkey anti-mouse; 1:500, A32787, ThermoFisher Scientific, Waltham, MA; Alex Fluor® 568 donkey anti-goat; 1:500, A11057, ThermoFisher Scientific, Waltham, MA). Retinal sections were mounted with ProLong®Gold Antifade Reagent with DAPI (#8961, Cell Signaling Technology Inc., Danvers, MA). Retinal sections were imaged using a Nikon A1R HD25 confocal microscope with a Plano Apo 20 × NA 0.75 objective and compiled and quantified using ImageJ software. KC and IP-10 labeling quantifications are the result of positive KC and IP-10 labeling within the superior, inner and deep vascular retinal plexi (n = 6 animals per group, each symbol in the plots represents the average of three retinal sections per animal). Cone arrestin labeling quantification is the result of positive cone arrestin labeling within the outer/inner segments and outer nuclear layer (n = 8 per group, 3 retinal sections quantified per animal).

### Multiplexed cytokine immunoassay

Whole retinal extract (excluding RPE, n = 4–9 animals per group) was flash frozen and lysed using a Bio-Plex cell lysis kit (Bio-Rad, Fort Worth, TX) according to manufacturer’s protocol, with the addition of one cOmplete mini (Roche, San Jose, CA) protease inhibitor per 5 mL of buffer. Lysates were placed on an end-over-end rotator for 20 min and centrifuged at 13.2kRPM. Protein concentrations were determined using a Pierce BCA Protein Assay (Thermo Fisher, Waltham, MA). Samples were normalized to a concentration of 0.75 μg in 37.5 μL of Milliplex® MAP Assay Buffer (EMD Millipore, Burlington, MA) because this loading fell within a linear range of detectable analytes^38^. Multiplexed cytokine quantification was conducted using the Milliplex® MAP Mouse Cytokine/Chemokine 32-Plex Kit (Eotaxin*, G-CSF, GM-CSF*, IFN-γ*, IL-1α, IL-1β*, IL-2, IL-3*, IL-4*, IL-5*, IL-6*, IL-7*, IL-9*, IL-10*, IL-12p40*, IL-12p70*, IL-13, IL-15*, IL-17*, IP-10, KC, LIF*, LIX*, MCP-1*, M-CSF*, MIG, MIP-1α, MIP-1β*, MIP-2, RANTES, TNF-α, and VEGF) (EMD Millipore) and read on a MAGPIX® system (Luminex, Austin, TX). Cytokines marked with an asterisk did not fall within a linear range of fluorescent intensity vs. protein concentration and were therefore excluded from the analysis.

### Masking and statistical analysis

Sample size was determined based on our previously reported data^16,17,19^. The observers who analyzed the data were blinded to the experimental procedures and were masked to the specific treatment group from which sampling arose. This included semi-automated marking of ERG a- and b-waves (Matlab 2021b, Mathworks, Natick, MA), image acquisition, semi-automated quantification of KC and IP-10 fluorescence labeling in retinal sagittal sections (ImageJ software). Statistical analyses were performed using Graphpad Prism 9.0.0 (San Diego, CA). Two-way ANOVAs dividing groups by exercise and light exposure to test the interactive effects of the independent variables and Tukey’s multiple comparison tests were performed on all visual and retinal functional data, ONL counts, cone photoreceptor counts, and fluorescence quantification and are presented as mean ± standard error of the mean (SEM). A Pearson correlation coefficient was computed to assess the linear relationship between individual animals KC and IP-10 expression, retinal function (scotopic a- and b-wave function and photopic b-wave function) and visual function (spatial frequency and contrast sensitivity). Multiplex cytokine data were analyzed using both univariate and multivariate techniques. For univariate analysis, active vs inactive samples were compared using an unpaired t-test with Welch’s correction to account for differing sample sizes among the groups. To account for co-variance among cytokines, we also conducted a partial least squares discriminant analysis (PLSDA) using the ropls package in R (Bioconductor^39,40^). To ensure the results of the PLSDA were robust to a subset of the samples (mice), we conducted a leave-n-out cross validation computing 100 iterations of PLSDA while each time leaving out four random samples (~ 15% of samples) then computed the standard deviation of loadings across all iterations.

All p-values lower than 0.05 were considered statistically significant. The ROUT method (with Q set to 1%) was used to detect outliers, of which none were detected.

## Results

### Voluntary exercise preserves retinal and visual function in I307N *Rho* induced mice

**Figure 2:**
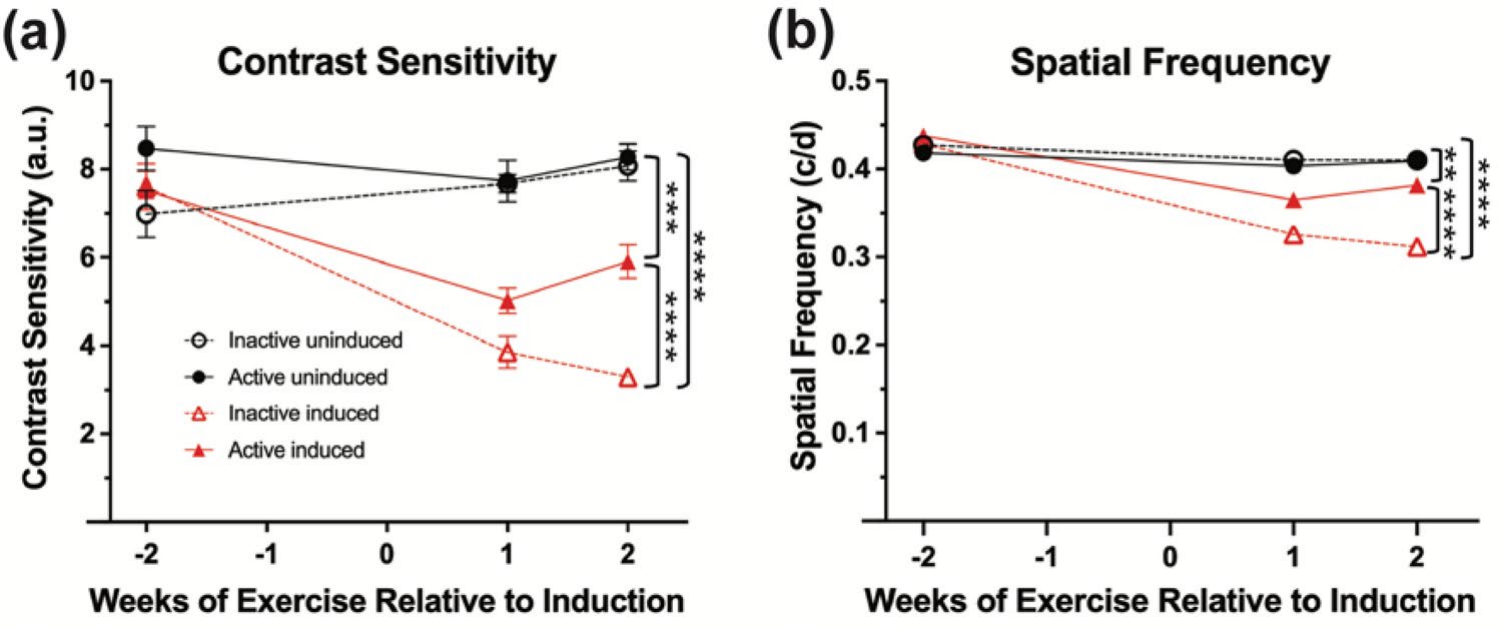
Contrast sensitivity and spatial frequency thresholds are preserved in active induced mice compared to inactive induced mice. Optomotor response (OMR) was performed at baseline and 1- and 2- weeks post-induction to measure visual function. At 1- and 2-weeks post induction, active induced mice had significantly greater contrast sensitivity (**a**) and spatial frequency (**b**) thresholds compared to inactive induced mice. N = 11–16 per group, **p < 0.01, ***p < 0.001, ****p < 0.0001. Values are mean ± SEM.

Electroretinography (ERG) was performed on all experimental groups to assess the effect of voluntary exercise on retinal function preservation in younger I307N *Rho* mice (Fig. 1a–e, Table 1). Similar to our previous work, active induced mice undergoing retinal degeneration had statistically significant preservation of retinal function with 1.44 × greater scotopic a-wave amplitudes (Fig. 1c, two-way ANOVA, F_(3, 126)_ = 32.12, p < 0.0001) and 1.42 × greater scotopic b-wave amplitudes (Fig. 1d, two-way ANOVA, F_(3, 126)_ = 28.06, p < 0.0001) and 1.31 × greater photopic b-wave amplitudes (Fig. 1e, two-way ANOVA, F_(3, 126)_ = 20.99, p < 0.0001) compared to inactive induced mice 2-weeks following light induction (Fig. 1, Table 1; two-way ANOVA with Tukey’s multiple comparison analysis was performed, n = 11–13 per group, average of both eyes). At 1- and 2- weeks post-induction, active induced mice had significantly greater visual function measured by contrast sensitivity (Fig. 2a; two-way ANOVA, F_(3, 144)_ = 39.13, p < 0.0001, n = 11–16 per group) and spatial frequency thresholds (Fig. 2b; two-way ANOVA, F_(3, 144)=_ 82.90, p < 0.0001, n = 11–16 per group) using OMR compared to inactive induced mice.

**Table 1:** Scotopic a-wave, scotopic b-wave and photopic b-wave ERG amplitude values to scotopic (2.1 log cd s/m^2^) and photopic (1.4 log cd s/m^2^) flash stimuli, respectively.

Experimental group	Scotopic a-wave (mean ± SEM)	Scotopic b-wave (mean ± SEM)	Photopic b-wave (mean ± SEM)
Inactive uninduced	313.2 μv ± 20.21	490.2 μv ± 35.13	145.2 μv ± 8.00
Active uninduced	253.1 μv ± 7.80	397.9 μv ± 21.71	142.4 μv ± 9.07
Inactive induced	71.4 μv ± 10.23	152.26 μv ± 27.91	61.6 μv ± 12.82
Active induced	181.1 μv ± 20.47	304.1 μv ± 19.99	101.2 μv ± 8.97
F ratio, p-value	F_(3,126)_ = 32.12, p < 0.0001	F_(3,126)_ = 28.06, p < 0.0001	F_(3,126)_ = 20.99, p < 0.0001

### Voluntary exercise conserved photoreceptor nuclei in active mice undergoing retinal degeneration

**Figure 3:**
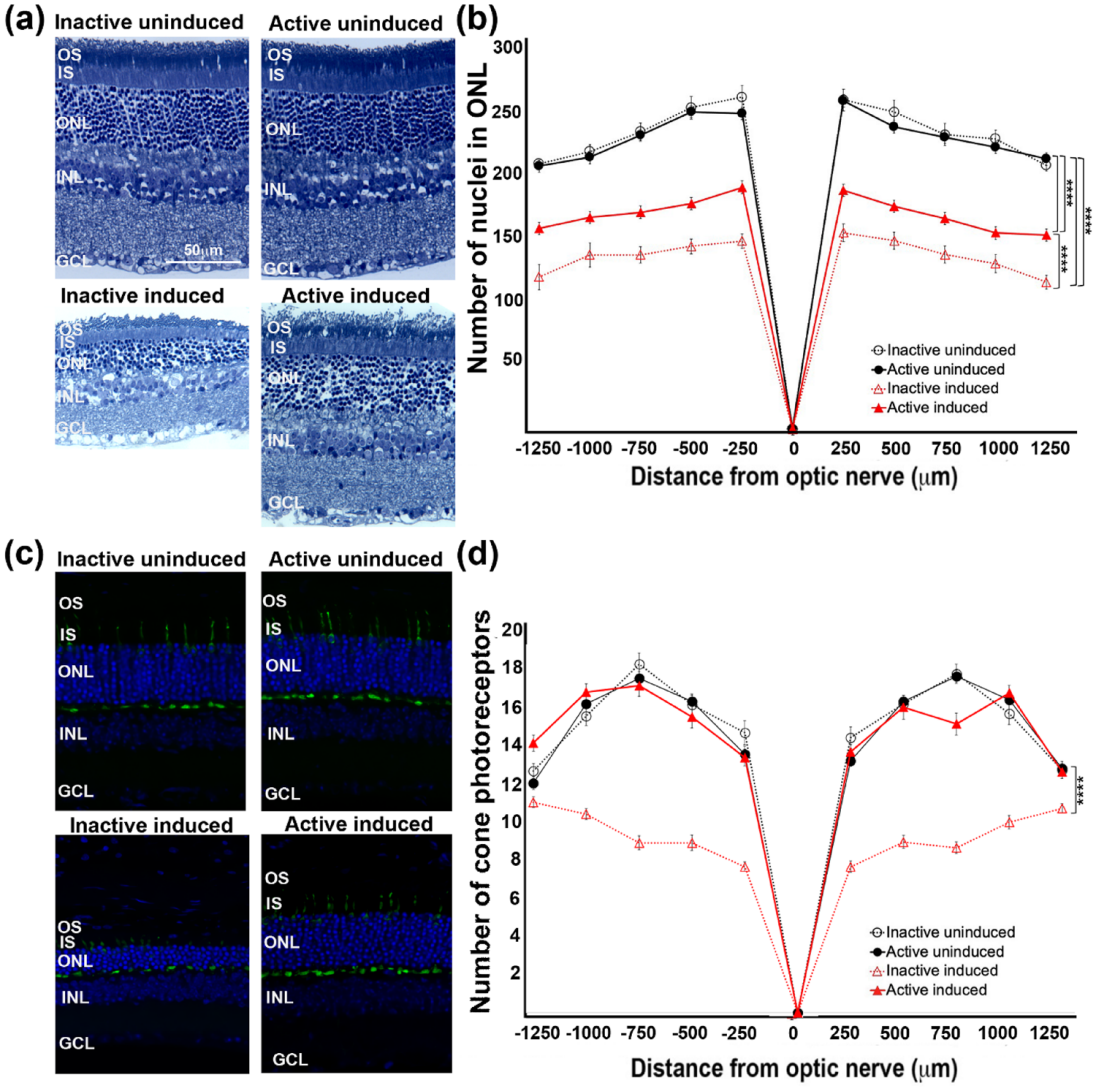
Active induced I307N *Rho* mice maintained photoreceptor nuclei. Retinal sections from all experimental groups (**a,c**) were used to quantify photoreceptor nuclei present in the outer nuclear layer (ONL). Morphometric analyses were constructed by plotting the quantification of ONL nuclei and cone photoreceptors by cone arrestin labeling as a function of position in the retina relative to the optic nerve (μm) spidergram (**b,d**). Although photoreceptor loss was evident in both induced groups, active induced mice had consistently greater numbers of outer nuclear layers when compared to inactive induced groups. Retinal layers are as follows: outer segment (OS), inner segment (IS), outer nuclear layer (ONL), inner nuclear layer (INL) and ganglion cell layer (GCL). N = 8 per group, 3 retinal sections quantified per animal, ****p < 0.0001. Values are mean ± SEM.

Outer nuclear layer (ONL) and cone photoreceptor quantifications were performed in retinal sagittal sections from all experimental groups. Consistent with our results measuring retinal and visual function, morphologic differences between active and inactive induced groups were apparent (Fig. 3a,c). Although photoreceptor loss was evident in both induced groups, active induced mice had significant preservation of photoreceptor nuclei compared to inactive induced mice (Fig. 3b; two-way ANOVA, F_(3, 864)_ = 771.9, p < 0.0001, n = 8 per group, 3 retinal sections quantified per animal) and cone photoreceptors (Fig. 3d; two-way ANOVA, F_(3, 864)_ = 300.3, p < 0.0001, n = 8 per group, 3 retinal sections quantified per animal).

### KC and IP-10 were lower in active mice

**Figure 4:**
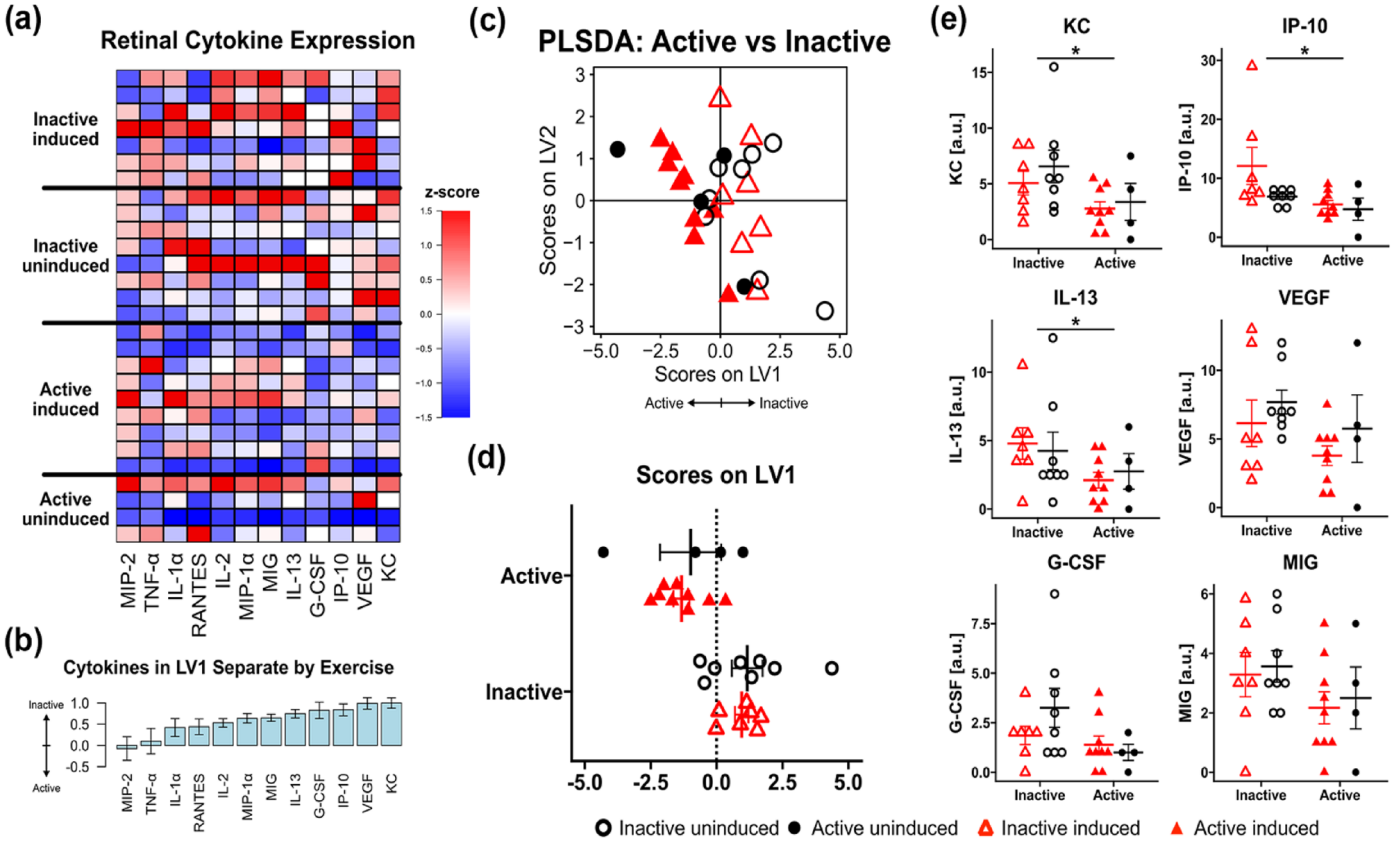
Cytokine expression elevated in inactive vs active mice. (**a**) Measured cytokines (columns, z-scored) from retinal extracts of all experimental animals (rows). (**b**) Partial least squares discriminant analysis (PLSDA) identified a profile of cytokines, LV1, correlated with active cases (negative) or inactive cases (positive). (**c,d**) LV1 separated inactive induced and uninduced cases to the right (unfilled shapes) and active induced and uninduced cases to the left (filled shapes). (**e**) Univariate analysis revealed significantly higher expression of KC, IP-10, and IL-13 in inactive vs active mice (*p < 0.05, unpaired t-test with Welch’s correction) and trending higher expression in inactive mice for VEGF, G-CSF, and MIG. N = 4–9 per group, each symbol in the plots represent retina from individual animals.

To investigate how exercise may influence retinal cytokine expression in an animal model of adRP, a multiplexed cytokine immunoassay was performed using retinal extract lysate from all experimental groups (Fig. 4a). To account for the multivariate nature of the data, we used a Partial Least Squares Discriminant Analysis (PLSDA) to identify a profile of cytokines associated with exercise (Fig. 4b). Although there was no clear separation among experimental groups based on induction, the PLSDA revealed an axis of cytokines, called latent variable 1 (LV1), that separated inactive animals to the right and active mice to the left (Fig. 4c,d). Top cytokines associated with inactivity included KC, IP-10, and IL-13 (Fig. 4b). Univariate analysis revealed significantly lower expression of keratinocyte derived chemokine (KC; t = 2.614, df = 24.07 p = 0.015), interferon gamma inducible protein-10 (IP-10; t = 2.300, df = 19.29, p = 0.0328), and interleukin-13 (IL-13; t = 2.128, df = 22.76, p = 0.0444) in active vs inactive groups (unpaired t-test with Welch’s correction, n = 4–9 per group, Fig. 4e). Additionally, cytokines with trending lower expression in active mice compared to inactive mice (p < 0.10, unpaired t-test with Welch’s correction) included vascular endothelial growth factor (VEGF; t = 2.046, df = 25.94, p = 0.0510), granulocyte-colony stimulating factor (G-CSF; t = 2.015, df = 21.71, p = 0.0564) and monokine induced by gamma interferon (MIG; t = 1.823, df = 25.38, p = 0.0801; Fig. 4e).

### KC and IP-10 labeling localized to retinal vasculature

**Figure 5:**
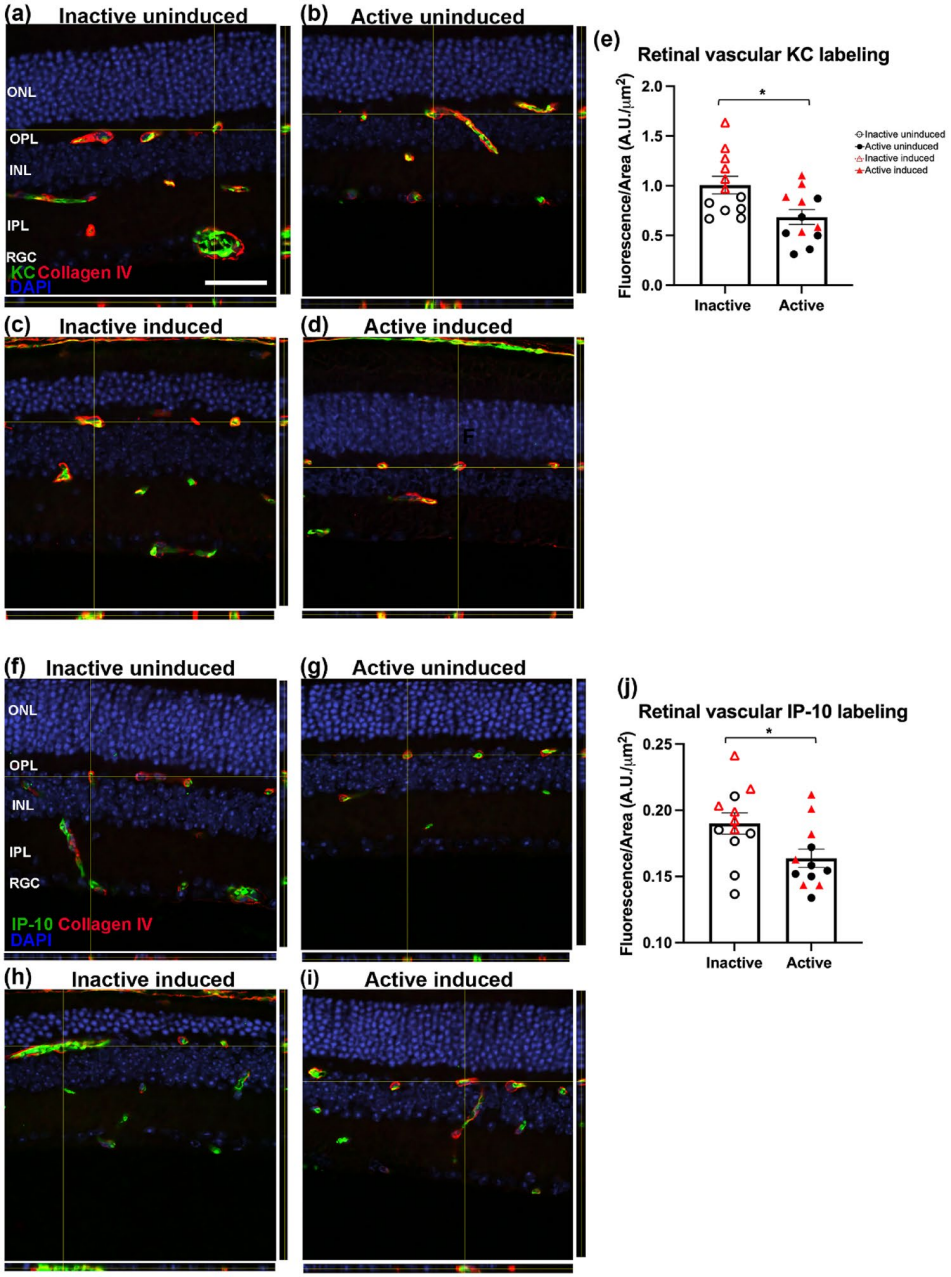
KC and IP-10 labeling localized to retinal vasculature. To determine the retinal cell type(s) expressing KC and IP-10, immunofluorescence on sagittal retinal sections from experimental groups was performed. Immunofluorescence revealed KC (**a–d**, green) and IP-10 (**f–i**, green) labeling localized to retinal vasculature as shown with co-labeling for collagen IV (**a–i**, red). We also observed inactive induced mice had a significant increase in both KC (**e**, p = 0.0110) and IP-10 (**j,** p = 0.0220) compared to uninduced mice. N = 6 animals per group, each symbol in the plots represents the average of three retinal sections per animal. KC and IP-10 labeling quantification are the result of positive KC and IP-10 labeling within the superior, inner and deep vascular plexi, *p < 0.05. Values are mean ± SEM.

In order to evaluate the retinal cell type(s) expressing KC and IP-10, immunofluorescence on sagittal retinal sections from experimental groups was performed. Immunofluorescence revealed KC and IP-10 labeling localized to retinal vasculature as shown with co-labeling with collagen IV (Fig. 5a–d,f–i). We also observed inactive animals had a significant increase in both KC (Fig. 5e, inactive: 1.006A.U./μm^2^ ± 0.0885; active: 0.685A.U./μm^2^ ± 0.0741; t = 2.783, df = 21.34, p = 0.0110, unpaired t-test with Welch’s correction; n = 6 animals per group, each symbol in the plots represents the average of three retinal sections per animal) and IP-10 (Fig. 5j, inactive: 0.190A.U./μm^2^ ± 0.0081; active: 0.164A.U./μm^2^ ± 0.00689; t = 2.470, df = 21.45 p = 0.0220, unpaired t-test with Welch’s correction; n = 6 animals per group, each symbol in the plots represents the average of three retinal sections per animal) labeling (Fluorescence/Area (A.U./μm^2^) compared to active groups.

### IP-10 expression has a moderate negative correlation with retinal and visual function assessments

**Figure 6:**
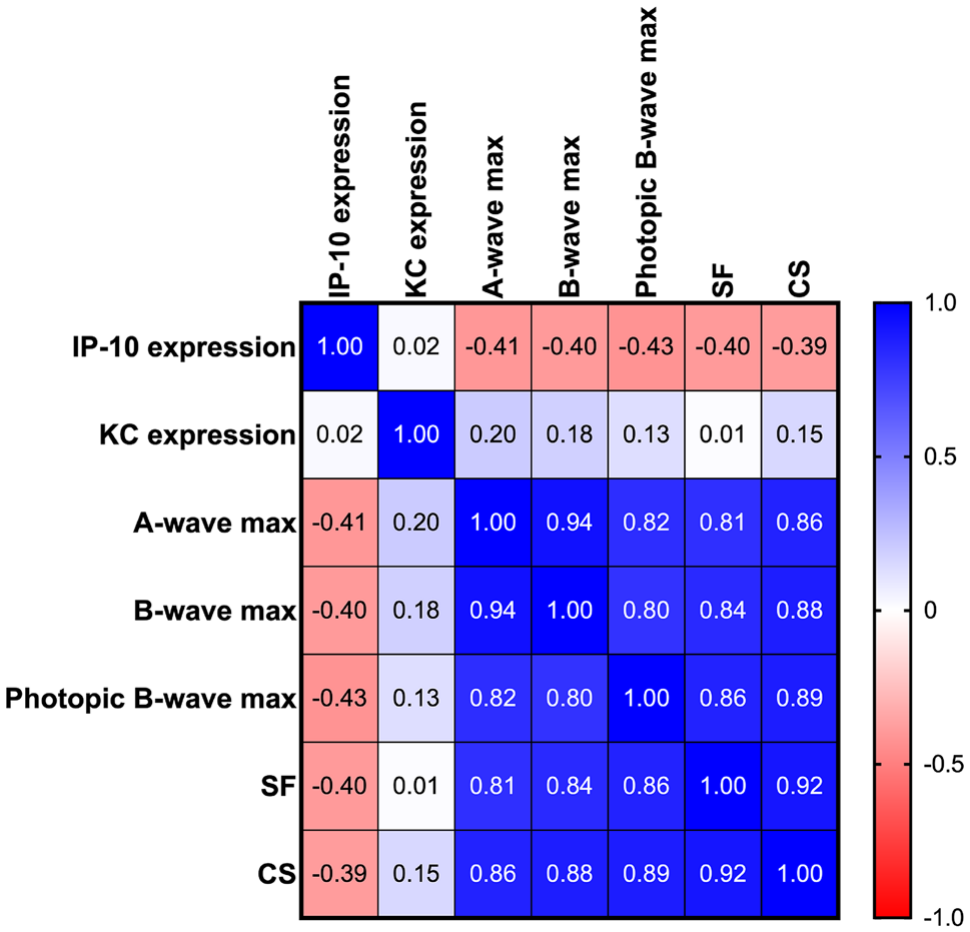
Moderate negative correlation of IP-10 expression associated with retinal and visual function assessments. Heatmap analyses was used to determine significant differences between individual animals for KC and IP-10 expression, retinal function (scotopic a- and b-wave function and photopic b-wave function) and visual function (spatial frequency; SF and contrast sensitivity; CS). IP-10 expression was found to have a moderate negative correlation with retinal (a-wave r = 0.41, p = 0.030; b-wave r =  − 0.40, p = 0.036; photopic b-wave r =  − 0.43, p = 0.024) and visual (SF r =  − 0.40, p = 0.036; CS r =  − 0.39, p = 0.041) function assessments. There was no significant correlation between KC expression and retinal (a-wave r = 0.201, p = 0.304; b-wave r = 0.182, p = 0.353; photopic b-wave r = 0.127, p = 0.520) and visual (SF r = 0.011, p = 0.958; CS r = 0.151, p = 0.442) function. N = 4–9 animals per group.

Pearson’s correlation coefficient shown as a heatmap was used to determine significant differences between individual animals KC and IP-10 expression, retinal function (scotopic a- and b-wave function and photopic b-wave function) and visual function (spatial frequency; SF and contrast sensitivity; CS, Fig. 6**,** n = 4–9 animals per group). IP-10 expression was found to have a moderate negative correlation with retinal (a-wave r = 0.41, p = 0.030; b-wave r =  − 0.40, p = 0.036; photopic b-wave r =  − 0.43, p = 0.024) and visual function assessments (SF r =  − 0.40, p = 0.036; CS r =  − 0.39, p = 0.041). There was no significant correlation between KC expression and retinal (a-wave r = 0.201, p = 0.304; b-wave r = 0.182, p = 0.353; photopic b-wave r = 0.127, p = 0.520) and visual function (SF r = 0.011, p = 0.958; CS r = 0.151, p = 0.442).

## Discussion

The protective effects of exercise for the prevention or treatment of neurodegenerative diseases are now well-documented in human and animal studies^11,13–15^. In this study, we tested whether an exercise regimen that protected against inherited retinal degeneration also altered retinal inflammatory responses. Determining how whole-body exercise alters the retinal cytokine network and inflammation in models of retinal degenerative diseases provides insight on how exercise-mediated interventions may halt and or slow vision loss. The eye, similar to the brain, is an immune privileged tissue. The retina reacts with a well-orchestrated and regulated multifaceted immune reaction in response to stress and insult^41^. Retinal degenerative diseases compromise the integrity of ocular immune privilege^42^. This results in overwhelming and non-resolving inflammation, ultimately moderating and driving disease progression.

In inherited retinal diseases, inflammatory responses have been generally considered as a secondary phenomenon associated with rod photoreceptor cell death, with the primary insult being induced by the causal genes (i.e., visual cycle protein mistrafficking, compromised photoreceptor structure integrity, inadequate RNA processing)^4,25^. However, the most effective neuroprotective treatments for adRP will also need to reduce this immune response to maintain maximal visual function. Inflammatory cytokines and chemokines elevated in RP are related to innate and acquired immunity^23,24^. In addition to maintaining retinal integrity, in this study we found both uninduced and induced active mice had significantly attenuated expression of cytokines KC and IP-10 and that their immunolabeling localized to retinal vasculature.

It is well-established that KC is a chemoattractant that recruits polymorphonuclear neutrophils to inflamed sites. In a study investigating the effects of treadmill exercise on cutaneous wound healing in aged mice, a significant reduction in KC expression was reported in dorsum tissue from exercised mice compared to inactive mice^43^. This study suggested that exercise not only accelerated the wound healing process, but also induced an anti-inflammatory response in the wound^43^. To date, retinal Müller glia, microglia, RPE and vascular endothelial cells have all been reported to have KC immunoreactivity^44,45^. Additionally, activation of CD40 expressed in retinal endothelial cells and Müller glia has been shown to promote KC production^45^. Recently, an in vitro study investigating the immunological reaction of murine microglia cells incubated with lipofuscin isolated from human RPE reported elevated KC levels in the cell culture supernatant^46^.

IP-10 is a pro-inflammatory cytokine that modulates angiogenesis and has been reported as a biomarker for age-related macular degeneration (AMD)^47–49^, another retinal disease characterized by photoreceptor degeneration. IP-10 was shown to be significantly elevated in all serum samples collected from subjects with different stages of both wet and dry AMD^48^. IP-10 labeling was abundant in the connective tissue matrix associated with choroidal neovascularization as well as neovascular endothelial cells in postmortem retinal tissues from AMD subjects^48^. Elevated expression of IP-10 and vascular growth factors VEGF and platelet derived growth factor (PDGF) have also been reported in the aqueous humor of AMD patients^50^.

In our study, we report diminished KC and IP-10 expression in retinal extract from active mice, both uninduced and induced compared to inactive groups. Furthermore, correlation coefficients revealed a moderate negative correlation between IP-10 expression and visual function outcome measurements. In retinal sections, KC and IP-10 were both localized to retinal vasculature through co-labeling with collagen IV. In future studies, analysis of retinal tissue at acute timepoints (12 h and 1, 3, and 5 days after induction) may provide further insight of the initial alterations exercise elicits on the retinal cytokine network during retinal degeneration in the I307N *Rho* mouse model. These studies could also explore the retinal and immune cell-types mediating exercise-induced cytokine response. Incorporating animal knockout models or antagonists of the relevant cytokines or upstream phospho-protein signaling pathways may also identify targeted mechanisms that can be rapidly translated based on currently available small molecules and antibody therapies^51,52^. Additionally, investigating other animal models of retinal disease, such as diabetic retinopathy and glaucoma, would provide insight on similarities and/or differences in exercise-induced retinal cytokine responses. Future studies could also investigate how exercise may modify retinal epigenetic mechanisms, altering retinal transcriptional patterns, and potentially altering causal genes^53,54^.

We demonstrated that voluntary running wheel exercise provided significant preservation of retinal and visual function as well as conservation of photoreceptor nuclei number in an inducible model of adRP, I307N *Rho*. Our study provides the first evidence that exercise significantly attenuates retinal KC and IP-10 protein levels in both healthy and degenerating retinas. We further determined that KC and IP-10 labeling localized to retinal vasculature in all experimental groups. Correlation analyses revealed IP-10 expression had a significant moderately negative association with all retinal and visual functional assays, whereas the relationship between KC expression and retinal functional measurements was not as robust. This study demonstrates that whole-body exercise can alter the retinal cytokine response in both healthy and degenerating retinas. These data provide evidence that supports exercise as a potential effective therapeutic strategy for inherited retinal degenerations, which currently have minimal treatment options.

## Data Availability

Although these data are not currently publicly available for sharing, requests for sharing can be sent to the Corresponding Author and will be evaluated on an individual basis.
